# Dietary Interventions, Supplements, and Plant-Derived Compounds for Adjunct Vitiligo Management: A Review of the Literature

**DOI:** 10.3390/nu17020357

**Published:** 2025-01-20

**Authors:** Michael J. Diaz, Jasmine T. Tran, Drake Rose, Aria Wei, Deepak Lakshmipathy, Shari R. Lipner

**Affiliations:** 1 College of Medicine, University of Florida, Gainesville, FL 32601, USA; 2 School of Medicine, Indiana University, Indianapolis, IN 46202, USA; 3 University of Michigan Medical School, Ann Arbor, MI 48109, USA; 4 School of Medicine, University of Texas Southwestern Medical Center, Dallas, TX 75390, USA; 5 Carle Illinois College of Medicine, University of Illinois Urbana-Champaign, Urbana, IL 61801, USA; 6 Department of Dermatology, Weill Cornell Medicine, New York City, NY 10021, USA

**Keywords:** vitiligo, diet, nutrition, autoimmune disease, convergence theory, supplementation, herbal medicine, natural products, macronutrients, micronutrients, vitamins, phytochemicals, plant-derived products

## Abstract

Vitiligo is a chronic autoimmune pigmentation disorder shaped by a complex interplay of genetic predispositions and environmental triggers. While conventional therapies—phototherapy, corticosteroids, and immunosuppressants—can be effective, their benefits are often partial and temporary, with recurrence common once treatment stops. As such, there is increasing interest in exploring complementary approaches that may offer a more sustainable impact. Emerging evidence suggests that macronutrient and micronutrient-level changes could be beneficial for managing progression and, in some cases, facilitating repigmentation. Antioxidant-rich foods, such as apples, green tea, Indian gooseberry, onions, and peppers, may help mitigate oxidative stress, while inflammatory foods, such as gluten and high-phenol nuts and berries, may exacerbate the condition. Certain supplements, including high-dose vitamin D, vitamin C, vitamin E, and selenium, may enhance phototherapy outcomes. Omega-3 and other unsaturated fatty acids, in addition to prebiotics and probiotics, are under active investigation for their roles in gut health and immune regulation. Notably, plant-derived compounds, i.e., *Ginkgo biloba*, have demonstrated promise in promoting repigmentation and managing disease progression. However, it must be emphasized that these nutritional interventions remain exploratory, and more research is needed to establish their efficacy, safety, and optimal usage before they can be recommended as part of a standard treatment regimen.

## 1. Introduction

Vitiligo is a chronic autoimmune skin condition characterized by melanocyte loss, resulting in the appearance of distinctive patches of skin depigmentation [1]. A pooled analysis of 71 studies estimated a global lifetime prevalence of clinician-adjudicated vitiligo of 0.36% (95% credible interval 0.24–0.54) [2], and a cross-sectional survey of the United States population (12/2019-03/2020, *n* = 40,888) suggested that >40% of adult vitiligo may be undiagnosed [3]. Central to the current understanding of vitiligo is the role of autoreactive cytotoxic CD8+ T cells, which interact with melanocytes, driving disease progression [4]. These T cells trigger the local production of IFN-γ, a key cytokine implicated in vitiligo pathology [5]. Additionally, IFN-γ-induced chemokines released by surrounding keratinocytes further amplify the immune response by recruiting more T cells to the affected skin, creating a self-perpetuating cycle [6].

**Figure 1:**
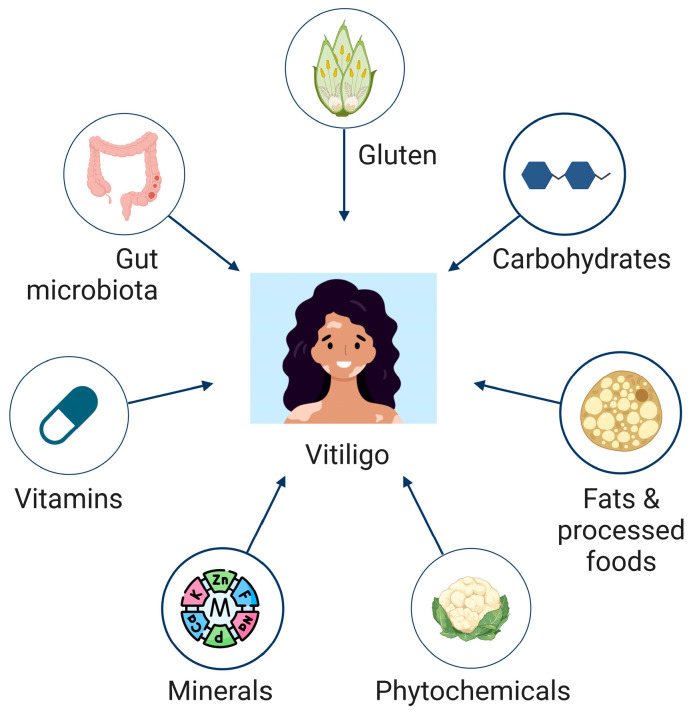
Illustration of nutrients and micronutrients with studied implications for vitiligo progression and management.

While topical and systemic treatments targeting IFN-γ signaling, e.g., JAK inhibitors, have shown clinical efficacy in vitiligo management [7,8,9,10,11], their benefits are often short-lived. Disease recurrence is common upon treatment cessation, largely due to the persistence of autoreactive resident memory T cells [12,13]. This therapeutic challenge has sparked interest in complementary and alternative medicine (CAM) strategies, including dietary modifications, supplementation, and herbal remedies, which are emerging as potential adjuncts to conventional therapies (Figure 1) [14,15,16,17]. These approaches aim to modulate immune responses, improve antioxidant defenses, and promote overall skin health, providing holistic options for patients with severe or treatment-resistant vitiligo.

Available evidence suggests that patients with vitiligo have decreased richness and diversity of their microbiota compared to healthy controls (*p* < 0.01) [18]. Alterations in the gut microbiota composition may modulate immune responses and inflammation [19]. In addition, a Mendelian randomization analysis supports a genetic association between antioxidant-rich beverages, including coffee (odds ratio [OR]: 0.17, 95% confidence interval [CI]: 0.07–0.37, *p* = 1.57 × 10^−5^), tea (OR: 0.99, 95% CI: 0.98–0.99, *p* = 5.66 × 10^−7^), and red wine (OR: 0.28, 95% CI: 0.08–1.00, *p* = 0.049), with reduced vitiligo risk [20]. The objective of this review is to provide a comprehensive update of nutrition-based management of vitiligo, particularly in patients with severe, treatment-resistant, or poorly responsive disease. This review highlights the potential of underexplored plant-derived compounds, including *Piperine* and *Nigella sativa*, as adjuncts in vitiligo management. While prior literature has focused broadly on dietary interventions, our work highlights emerging evidence on microbiome-targeted approaches and the role of specific supplements that modulate oxidative stress and immune responses. By synthesizing findings from recent studies, this review aims to provide a more up-to-date, comprehensive overview that reflects the evolving landscape of adjunct therapies for vitiligo management. While promising, these approaches remain investigational, and these interventions should not be considered definitive treatment recommendations until further high-quality clinical trials confirm their safety and efficacy. Long-term studies on the sustainability of repigmentation after discontinuation of dietary interventions are sparse. Preliminary evidence suggests that antioxidant-rich diets and plant-derived compounds may delay relapse by modulating oxidative stress and immune activity. However, prospective trials with extended follow-up are needed to confirm these potential benefits.

## 2. Pathogenesis of Vitiligo

### 2.1. Convergence Theory: A Unified Framework

**Figure 2:**
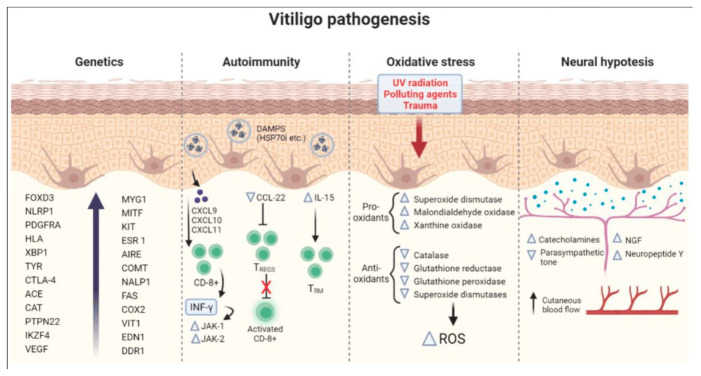
The convergence theory for vitiligo. Adapted from [21].

The convergence theory posits that vitiligo results from a multifactorial interplay between autoimmunity, genetic predisposition, oxidative stress, and neurogenic factors (Figure 2) [21,22,23]. This theory recognizes that autoimmune mechanisms drive the immune system’s erroneous targeting of melanocytes, while genetic susceptibility lays the groundwork for heightened vulnerability. Environmental triggers, i.e., oxidative stress and neurogenic dysfunction, further exacerbate melanocyte loss.

This theory also accommodates secondary influences, such as trauma, i.e., the Koebner phenomenon; cytokine signaling; viral agents; and intrinsic melanocyte defects, highlighting their potential roles in disease initiation and progression. By integrating these interconnected pathways, the convergence theory provides a comprehensive lens to understand vitiligo and informs the need for multifaceted therapeutic approaches.

### 2.2. Autoimmunity

The autoimmune hypothesis suggests that vitiligo stems from the immune system mistakenly destroying melanocytes and causing loss of pigment in affected areas [24]. Support for this hypothesis stems from the discovery that patients with vitiligo often have comorbid autoimmune diseases. A review of patients with vitiligo (*n* = 1098) registered in the Henry Ford Health System reported a higher prevalence of thyroid disease (12.9%, *p* < 0.001), alopecia areata (3.8%, *p* < 0.001), pernicious anemia (0.5%, *p* = 0.007), systemic lupus erythematosus (0.3%, *p* = 0.048), Guillain-Barre syndrome (0.3%, *p* = 0.001), linear morphea (0.2%, *p* = 0.001), myasthenia gravis (0.2%, *p* = 0.002), and Sjögren syndrome (0.2%, *p* = 0.011) compared to the general population [25]. Similarly, a retrospective review showed a higher-than-population prevalence of hypothyroidism (12.0%, *p* < 0.01) and pernicious anemia (1.3%, *p* < 0.01) in vitiligo patients (*n* = 300) [26]. In addition, within 12 to 24 weeks, 91.8% (*n* = 408) of analyzed vitiligo patients (*n* = 444) treated with low-dose oral mini-pulse dexamethasone therapy experienced cessation in the development of new lesions and expansion of existing ones with minimal side effects [27]. Twice daily application of topical calcineurin inhibitors tacrolimus (ointment 0.03%) and pimecrolimus (cream 1%) for 6 months each achieved a 100% response rate in 42 infants, with reported efficacy rates (>50% repigmentation) of 69.6% and 65.2%, respectively [28].

### 2.3. Oxidative Stress

Vitiligo may also be induced by oxidative stress, which is characterized by states of reactive oxygen species (ROS) overproduction, low levels of antioxidants, and disturbed antioxidant pathways and polymorphisms of ROS-associated genes [29]. Increased oxidative stress within the skin microenvironment can cause melanocyte damage and melanogenesis disruption, activation of pro-inflammatory pathways, and depigmentation in affected regions. In a clinical case-control study measuring indicators of oxidative stress, vitiligo patients (*n* = 16) had higher levels of superoxide dismutase (*p* < 0.001) and plasma malondialdehyde (*p* < 0.001) and lower levels of catalase (*p* < 0.05) and glucose 6-phosphate dehydrogenase (*p* < 0.001) compared to healthy controls (*n* = 16) [30]. Similarly, a cross-sectional analysis reported that patients with active vitiligo (*n* = 39) had higher generation of ROS in erythrocytes (*p* < 0.01), MDA levels (*p* < 0.05), and oxidatively damaged guanine species (*p* < 0.05) compared to healthy controls (*n* = 54) [31]. A meta-analysis of four RCTs (*n* = 91) found that treatment of vitiligo patients with a combination antioxidant supplementation and phototherapy increased ≥50% repigmentation of all lesions in an individual patient compared to phototherapy alone (relative risk: 1.87, 95% confidence interval: 1.10–3.17) [32].

### 2.4. Neurogenic Factors

Disruption of neuro-immuno-cutaneous interactions, including neuropeptide signaling, may contribute to the melanocyte loss in vitiligo lesions. To assess the possible role of neuropeptide-Y (NPY) on vitiligo development, NPY levels were measured in tissue fluid collected from skin lesions of vitiligo patients (*n* = 47) and uninvolved skin from healthy controls (*n* = 32). Patients with local (*p* < 0.01) and segmental (*p* < 0.05) vitiligo types had higher NPY levels than controls, suggesting that NPY may be involved in the pathogenesis of specific vitiligo subtypes [33]. In addition, there are increased allele frequencies for NPY − 399T/C (*n* = 454, *p* < 0.0001) and +1128T/C (*n* = 575, *p* < 0.0001) polymorphisms in vitiligo patients compared to controls (*n* = 1226, 1279, respectively) [34]. The neuropeptide, alpha-melanotropin (α-MSH), has a role in melanin production and host defense. In a study investigating melanotropins and their role in vitiligo, patients with the condition (*n* = 40) had significantly lower median α-MSH levels (6.4 pmol/L [5.2; 11.3]) compared to healthy controls (*n* = 40; 11.4 pmol/L [8.6; 13.4]) (*p* = 0.01) [35].

Neuroendocrine factors have been implicated in vitiligo pathogenesis. Increases in plasma levels of epinephrine (*p* < 0.001), norepinephrine (*p* < 0.001), dopamine (*p* < 0.001), serotonin (*p* < 0.001), and melatonin (*p* < 0.001) were reported in patients with acrofacial vitiligo (*n* = 60) compared to healthy controls (*n* = 40) and in those with active vitiligo compared to stable vitiligo (*p* < 0.001) [36]. Brain-derived neurotrophic factor (BDNF), which is vital for synaptic plasticity and memory processes, has been implicated in cognition and neuropsychiatric disorders [37,38]. Mean serum BDNF values were higher (*p*-0.007) in healthy controls (*n* = 58) compared with vitiligo patients (*n* = 57) in an age- and sex-matched case-control study [38].

### 2.5. Role of Genetic Predisposition

Genetic susceptibility plays a significant role in vitiligo development, as evidenced by the high prevalence of vitiligo in close relatives and the high disease heritability [39,40]. In a 2016 genome-wide association study, 23 loci achieved genome-wide significance (*p* < 5 × 10^−8^), and 7 loci achieved suggestive significance (*p* < 1 × 10^−5^) for vitiligo in patients of European ancestry (4680 cases, 39,586 controls) [41]. Multiple susceptibility genes were identified, including those involved in immune regulation, melanocyte function, and other autoimmune diseases. HLA genes, most often attributed with structural specificity during self-antigen presentation, are often implicated. The HLA-DRB1/DQA1 locus was more prevalent (*p* = 2.07 × 10^−5^) in multiplex-affected families (*n* = 444) with at least two relatives with generalized vitiligo compared to simplex cases with no family history of vitiligo, suggesting that HLA-related adaptive immune responses may be especially important to vitiligo pathobiology in multiplex-affected families [42]. Similarly, the SRY-box (SOX) transcription factors have been previously purported as autoantigens, and a microarray analysis of differentially expressed genes indicated SOX10 downregulation in the blood of vitiligo patients (*n* = 13) compared to controls (*n* = 9), suggesting involvement of premelanosome proteins in the process of vitiligo [43].

More specifically, polymorphism in the GZMB gene, which encodes the enzyme Granzyme B and is involved in cytotoxic T cell-induced apoptosis, may be associated with autoimmune disease, including vitiligo. In patients with Chinese Han ancestry (*n* = 3120), genotyping of 15 GZMB SNPs showed that SNP rs8192917 was associated with vitiligo disease status (OR = 1.39, *p* = 1.92 × 10^−8^) [44].

In support of these findings, an *in silico* analysis in a Korean population (*n* = 249) with non-segmental vitiligo identified an association between the GZMB SNP rs8192917 (OR = 1.43, *p* = 0.007) and NSV development. Additionally, SNPs rs2236338 (OR = 1.44, *p* = 0.007), rs11539752 (OR = 1.56, *p* = 0.001), rs10909625 (OR = 1.49, *p* = 0.002), and rs8192917 (OR = 1.43, *p* = 0.007) were also associated with disease risk [45].

### 2.6. Recent Insights

Recent studies have highlighted the central role of autoreactive T cells, the dysregulation of immune pathways, and cytokine signaling in vitiligo pathogenesis [46,47,48,49]. Elevated levels of type-1 innate NK and ILC1 cells (*p* = 0.03) were observed in both the blood and non-lesional skin of vitiligo patients (*n* = 11) in contrast to healthy individuals (*n* = 6) [50]. IFNγ, generated by NK and ILC1 cells, was associated with higher levels of CXCL9 (*p* = 0.018) and CXCL10 (*p* = 0.02) proteins in the supernatant of vitiligo melanocytes, equivalent to an average 4- to 5-fold increase in chemokine production compared to controls. Similar findings in a transcriptome analysis showed that genes upregulated in T cells within vitiligo-affected skin (*n* = 8), such as IFN-γ, TNF-α, and IL-13, are linked to cytokine-mediated signaling, impacting inflammatory chemokine expression in melanocytes and keratinocytes (P-adj < 0.05) [51]. Together, these findings suggest that cytokines and T cells enhance chemokine expression to promote epidermal inflammation in vitiligo melanocytes.

Genetic studies have identified additional RNA properties associated with vitiligo susceptibility and disease progression. Single-cell transcriptomic analysis showed differences (*p* < 0.05) in cell type proportions between skin samples from vitiligo patients (*n* = 10) and healthy donors (*n* = 5), with melanocyte-specific RNA-binding protein (RBP) genes increased in the apoptosis and immune-related pathways of patients with vitiligo [52]. Knockdown of the RBP gene SLC3A2, a downregulated gene in vitiligo patients, was associated with decreased melanocyte proliferation (*p* < 0.001) and increased apoptosis (*p* < 0.001). Additionally, compared to controls, skin samples of patients with vitiligo showed 64 significantly dysregulated circRNA transcripts (*p* < 0.05) that are associated with metabolic pathways, including ubiquitin-mediated proteolysis, endocytosis and RNA degradation, and Jak-STAT signaling [53]. Thus, the circRNA-miRNA-mRNA network regulates melanocyte functions and is closely linked to melanocyte metabolism.

In addition to genetic and RNA-related mechanisms, dysregulated neuroendocrine signaling within the cutaneous microenvironment plays a pivotal role in melanocyte damage in vitiligo [54,55]. Elevated catecholamine levels, including epinephrine, norepinephrine, and dopamine (*p* = 0.035, 0.024, and 0.006, respectively), have been detected in perilesional skin biopsies from vitiligo patients (*n* = 30) compared to controls (*n* = 15), potentially exacerbating cellular stress and immune signaling [56]. However, these catecholamines were not elevated in plasma samples, underscoring the localized nature of these changes. Catalase gene expression was significantly upregulated in perilesional skin compared to unaffected sites (*p* = 0.001) and healthy controls (*p* = 0.037), suggesting an adaptive response to neutralize ROS.

## 3. Diet

Several dietary strategies have been explored for their potential to influence the management of vitiligo through mechanisms such as immune modulation, oxidative stress reduction, and gut microbiome balance. These strategies, along with key findings and studied populations, are summarized in Table 1.

### 3.1. High-Fat Foods

Specific dietary fats, particularly polyunsaturated fatty acids (PUFAs), influence immune function and inflammation [57,58]. A case-control study found that vitiligo patients (*n* = 100) consumed more saturated fatty acid (SFA) and less eicosapentaenoic acid (*p* = 0.001) and docosahexaenoic acid (*p* = 0.004) than control participants (*n* = 110) [59]. Increased total fat intake was also associated with increased risk of vitiligo (OR = 3.33, *p* = 0.01). Furthermore, a case-control study found that vitiligo patients (*n* = 60) had higher body mass index compared to age and sex-matched controls (*n* = 60) (*p* = 0.021), with an association between elevated intakes of fats and oils and a high Vitiligo Area Scoring Index (VASI) of 12.5 on the trunk (*p* = 0.041) [60]. In a study assessing serum levels of fatty acids in vitiligo patients (*n* = 48) and healthy individuals (*n* = 28), vitiligo patients had elevated levels of alpha-linolenic acid (ALA) and decreased levels of arachidonic acid (ARA), arachidic acid (AA), and behenic acid (*p*< 0.05) [61].

### 3.2. Sugar and Refined Carbohydrates

High carbohydrate/low protein diets can induce clinically detectable dysregulated autophagy, a self-destructive mechanism triggered by prolonged starvation, protein deficiency, and lack of micronutrients. This autophagy may underline the pathogenesis for vitiligo. In a retrospective study, vitiligo patients were categorized based on disease stage and dietary habits [62]. Both early-stage (within 6 months) vitiligo patients (*n* = 20) and those with stabilized disease (more advanced, stable condition) (*n* = 10) showed improvement in VASI at 6 months and 1-year intervals following conventional therapies combined with a low carb/high protein diet (*p* = 0.013 and *p* = 0.001, respectively). Similarly, a different multivariate logistic regression analysis showed that high consumption of cereals (*n* = 34) was correlated with elevated VASI on the upper limbs (*p* < 0.05) [60]. Excessive intake of refined grains was linked to rapid increases in plasma glucose levels, resulting in ROS generation through activation of the protein kinase C, hexosamine, and sorbitol pathways.

A two-sample mendelian randomization study showed that mannitol, pyruvate, and 1,5-anhydroglucitol are significantly associated with vitiligo by affecting immune cell function [63]. Additionally, random blood sugar analysis of vitiligo patients (*n* = 125) found higher levels in patients 31–40, 41–50, and >50 years (151.10 mg/dL, 197.50 mg/dL, and 210.00 mg/dL, respectively) compared to controls (105.30 mg/dL, 125.00 mg/dL, and 120.75 mg/dL, respectively) (*p* < 0.01 for all) [64]. Notably, vitiligo and type 1 diabetes mellitus share a similar pathogenesis involving autoreactive cytotoxic T-cell-mediated destruction [65].

### 3.3. Gluten

Case reports suggest that a gluten-free diet (GFD) may offer benefits for vitiligo patients, particularly those unresponsive to conventional therapies [66]. In one case, a 22-year-old Asian female with acrofacial vitiligo who had previously failed treatment with topical tacrolimus and calcipotriene, who then received oral dapsone 100 mg t.i.d. and phototherapy, experienced repigmentation within one month of adopting a GFD, which continued to improve for a successive 3 months [67]. In addition, a 9-year-old girl with vitiligo of the face, trunk, and limbs (diagnosed at age 6 y) and celiac disease experienced progressive repigmentation following a GFD over three years without conventional therapy, with sustained pigmentation maintenance seven years post-initiation of the GFD [68]. She had previously had no response to treatment by PUVA (9 months), topical prednicarbate 0.25% (6 months), and topical pimecrolimus (1 year). Although vitiligo and celiac disease share some autoimmune mechanisms and appear associated in observational data [66,69,70], powered prospective studies are needed to determine the broader applicability of GFD as a standalone dietary strategy in vitiligo management.

### 3.4. Impact of Gut Microbiota

Recent insights have highlighted the critical role of the gut microbiome in keeping immune homeostasis and in the development of autoimmune diseases, including vitiligo. A case-control study of 10 patients with stable non-segmental vitiligo and 10 healthy controls reported decreased richness and diversity of gut microbiota in the stool of vitiligo subjects (*p* < 0.01) [18]. Additionally, there was decreased gut microbial α-diversity, primarily due to differences in distinct taxa and phylogenetic diversity (observed amplicon sequence variants, *p* = 0.005; Faith’s phylogenetic diversity H = 5.851, *p* = 0.006), rather than microbial richness and evenness (Shannon index, *p* = 0.115). Similarly, a reduction in the α-diversity of the intestinal microbiome of 25 patients with advanced non-segmental vitiligo was found compared to 25 age-, sex-, and body mass index-matched health controls (Wilcoxon rank sum test, Shannon index *p* = 0.011, and Simpson index *p* = 0.002), which was accompanied by alterations in the relative abundance of specific bacteria, i.e., decreased Staphylococcus thermophiles and increased Bacteroides fragilis [71]. Of note, a separate assessment of gut microbial α-diversity by 16S rRNA sequencing in 30 patients with advanced non-segmental vitiligo (mean age 37.2 years) and 30 controls matched by age, sex, body mass index, and dietary habits (mean age 35.2 years) demonstrated increased diversity of the vitiligo gut microbiome (Kruskal–Wallis pairwise test, Shannon index *p* = 0.014, and Simpson index *p* = 0.027) [72]. A decreased Bacteroidetes–Firmicutes ratio (*p* < 0.05) was also observed for vitiligo patients (1.6:1) compared to healthy controls (2.3:1), which corroborates the significantly higher Firmicutes–Bacteroidota ratio observed in 32 patients with unstable non-segmental vitiligo (mean age 25.5 years) compared to 27 healthy controls (mean age 25.3 years) (*p* < 0.05) [72,73].

Given the protective role of Bacteroidetes in the gut of vitiligo mouse models and its association to the prevention of skin depigmentation, its relative composition in vitiligo patients may provide further insights into the pathogenesis of vitiligo [73]. This notion is supported by evidence showing that antibiotic treatment, which alters both skin and gut microbiomes, can lead to vitiligo development by reducing microbial diversity and enhancing B-cell and T-cell activity, ultimately resulting in melanocyte destruction [74]. Since recent evidence demonstrates the importance of the gut microbiome, and to acknowledge the variability in gut microbiome composition among patients, individualized approaches leveraging microbiome sequencing and personalized probiotic recommendations may be beneficial.

## 4. Nutrients

Nutritional interventions, including vitamins, trace elements, amino acids, and antioxidant supplements, have garnered significant interest for their potential role in mitigating oxidative stress, immune dysfunction, and melanocyte damage in vitiligo. Key supplements, their mechanisms of action, and the levels of evidence supporting or dissuading their use are summarized in Table 2.

### 4.1. Vitamins

Several studies have demonstrated an association between vitiligo and vitamin deficiencies. An Egyptian cross-sectional study of patients ages 14–65 y found that non-segmental vitiligo was linked to lower serum vitamin D levels by an enzyme-linked immunosorbent assay versus controls (*n* 30 vs. 40) (*p* < 0.001) [75]. A case-control study including 150 vitiligo patients (mean age 30.6 y, 40% female) showed that males (*p* = 0.01), younger individuals (*p* = 0.01), and those not undergoing UV treatment (*p* = 0.01) exhibited lower vitamin D levels compared to age- and gender-matched subjects without vitiligo [76]. The literature presents mixed findings concerning vitamins C and E. While a comprehensive meta-analysis including 570 vitiligo cases and 580 controls found no significant differences in serum levels of vitamins C and E between groups, contrasting results have been reported in smaller-scale studies [77,78].

Given the supported correlation between low vitamin D levels and vitiligo, there has been growing interest surrounding vitamin D supplementation in this patient population [79]. A pilot study evaluating the impact of daily, high-dose oral vitamin D supplements (35,000 IU per day for six months) in vitamin D-deficient adult vitiligo patients reported 25–75% pigmentation in 88% (14/16) [80]. Similarly, a sex- and age-matched prospective study of 14 children with vitiligo ages 6–17 years who were also vitamin D deficient reported that vitamin D supplementation, coupled with topical tacrolimus, resulted in decreases in depigmented areas over the course of six months (*p* < 0.001) relative to those receiving tacrolimus alone [81]. In contrast, a clinical pilot randomized study conducted in Iran and a randomized controlled trial performed in Mexico (NCT04872257) on patients of all ages (with and without pre-existing vitamin D deficiencies) found that vitamin D supplementation offered no additional repigmentation benefit in vitiligo patients when used alongside standard narrowband ultraviolet B phototherapy [82,83]. Additional prospective studies are needed to determine the utility of vitamin D supplementation in vitiligo patients.

### 4.2. Trace Elements

Recent studies and meta-analyses have highlighted the potential role of trace elements such as zinc, copper, and selenium in the etiology and pathogenesis of vitiligo. A meta-analysis of 41 studies (1970–2022) reported that vitiligo patients (*n* = 3353) had lower serum zinc (SMD = −0.86, 95% CI: −1.19 to −0.52, *p* < 0.0001) and copper levels (SMD = −0.50, 95% CI: −0.91 to −0.10, *p* < 0.0001) compared to controls (*n* = 10,638), with elevated serum selenium levels (SMD = 0.23, 95% CI: 0.58 to 1.04, *p* < 0.0001) [84].

Zinc plays a critical role in cutaneous homeostasis, immune response modulation, and activity of antioxidant enzymes, with deficiencies potentially worsening melanocyte damage due to increased oxidative stress [85,86]. Copper, a key cofactor for tyrosinase, directly influences melanogenesis; its depletion may impair melanin synthesis, contributing to hypopigmentation in vitiligo. An Indian hospital-based, cross-sectional study of 60 clinically diagnosed vitiligo cases (mean age 42 years) and 60 age- and sex-matched controls measured a significant positive correlation between lesional and non-lesional Cu levels of vitiligo patients (*p* < 0.05), while mean serum levels remained similar [87]. In addition, a case-control study of 100 vitiligo cases (mean age 26.1 years, 40% female) and 60 healthy controls (mean age 19.2 years, 55% female) reported lower mean serum Zn and higher Cu levels in vitiligo patients compared to controls (*p* < 0.001), underscoring the complex roles these elements may play in melanocyte health and disease progression [88]. Further research is essential to clarify the role of Cu and Zn and to develop evidence-based guidelines for potential use in clinical practice.

Selenium, essential for glutathione peroxidase activity, shields cells from free radical damage; elevated levels might reflect compensatory antioxidant responses, though excessive selenium may disrupt redox balance and affect melanocyte viability. Interestingly, a few studies have reported lower selenium levels in vitiligo patients, particularly among Asian populations (*p* < 0.05) [89,90,91]. These findings suggest that selenium supplementation strategies may require demographic tailoring.

### 4.3. Amino Acids

Phenylalanine, an essential α-amino acid, is a precursor for cutaneous pigment melanin that has been used therapeutically for the treatment of vitiligo [92,93,94]. In an open trial (*n* = 149, 18 months) and a double-blind trial (*n* = 32, 6 months), phenylalanine as an adjunct therapy with UVA treatment demonstrated repigmentation improvements, with responses up to 77% and 60%, respectively, though higher doses did not appear to enhance outcomes (optimal L-Phe < 50 mg/kg/day) [95]. In addition, 10% phenylalanine gel resulted in a 24% improvement in mean VASI score compared to baseline (0.237 vs. 0.312) in a sample of 37 patients with limited nonsegmental vitiligo (mean age 38 y, 56.8% female, 56.8% recent-onset vitiligo [<12 months]), with the greatest improvements seen for head and neck lesions (36.6%) [96]. No adverse effects were noted. Overall, phenylalanine is generally well-tolerated, but patients may experience gastrointestinal discomfort. Comparison with other amino acids could better clarify its role in repigmentation therapy.

Moreover, an Iranian metabolomics study in non-segmental vitiligo patients (*n* = 31) found that plasma levels of cysteine (*p* = 2 × 10^−9^), glutamic acid (*p* = 0.007), and proline (*p* = 3 × 10^−4^) were elevated, while arginine (*p* = 5 × 10^−7^), lysine (*p* = 5 × 10^−7^), ornithine (*p* = 1 × 10^−6^), glycine (*p* = 0.02), and histidine (*p* = 0.006) were decreased compared to healthy controls (*n* = 34) [97]. In an analysis of the receiver operating characteristic curves, cysteine and lysine together emerged as a best-candidate disease biomarker (0.96 AUC).

### 4.4. Unsaturated Fatty Acids

Current literature surrounding unsaturated fatty acid supplements and vitiligo is scarce. A case-control study reported a relationship (*p* < 0.05) between fatty acid metabolic pathways and metabolic profiles of vitiligo patients (*n* = 30), showing that metabolites found in the serum of vitiligo patients are also involved in fatty acid metabolism pathways; however, information regarding therapeutic targets was not reported [72]. Higher alpha-linolenic acid levels and lower arachidonic acid levels were found in vitiligo serum samples of 48 patients compared to the samples from 28 age- and gender-matched healthy controls (*p* < 0.05) [61]. Given that alpha-linoleic acid and arachidonic acid are both polyunsaturated fatty acids, the role of unsaturated fatty acid supplements in treating vitiligo is unclear. A comparative study (*n* = 16) examining oxidative stress biomarkers in the blood of vitiligo patients before and after phototherapy combined with an antioxidant supplement containing fatty acids and an RCT (*n* = 35) evaluating the clinical effectiveness of phototherapy and antioxidant pool oral supplementation containing fatty acids to reduce oxidative stress-induced damage in nonsegmental vitiligo patients provided more clear support for unsaturated fatty acid supplement use [98,99]. Both reported significantly (*p* < 0.05) improved repigmentation in patients treated with phototherapy and combination oral antioxidant supplements containing polyunsaturated fatty acids versus phototherapy alone.

### 4.5. Antioxidant Pool

Antioxidants either limit the production of free radicals or facilitate their clearance and thus have garnered attention as a possible treatment for vitiligo [20,100,101,102,103,104]. These supplements include a broad variety of vitamins, trace minerals, fatty acids, and myriad other bioactive compounds [78]. In a randomized, double-blind, placebo-controlled trial of narrowband UVB treatment of nonsegmental vitiligo (*n* = 35), supplementation with an antioxidant pool containing alpha-lipoic acid (50 mg), vitamin C (50 mg), vitamin E (20 mg), polyunsaturated fatty acids (12%), and cysteine monohydrate (50 mg) 2 months before and 6 months during therapy significantly increased treatment effectiveness (>75% repigmentation attained by 47% versus 18%, *p* < 0.05) and decreased the production of reactive oxygen species up to 60% versus baseline (*p* < 0.02) [99]. Combination antioxidant supplements derived from herbal medicines have also shown clinical utility. An Italy-based controlled trial analyzed the benefit of an oral supplement containing *P. emblica* (100 mg), vitamin E (4.7 mg), and carotenoids (10 mg) taken three times daily for 6 months in an adult population with nonsegmental vitiligo receiving concomitant therapy (93.1% corticosteroids) [105]. Compared to subjects who did not receive treatment (mean age 36.2 years, mean age at onset 27.1 y, *n* = 65), the treatment population (mean age 38.0 years, mean age at onset 29.2 years, *n* = 65) experienced significant mild (1–25%) repigmentation in the head and neck region (*p* = 0.019). Additionally, the control population had increased lesion growth (*p* = 0.039) and inflammation (*p* = 0.002). More research is still needed to determine long-term safety and synergistic effects of high-dose antioxidant supplementation.

### 4.6. Probiotics and Prebiotics

Interest in the use of probiotic and prebiotic supplements in vitiligo patients is based on data from the gut–skin axis in autoimmune and inflammatory skin diseases. There are reported associations between gut dysbiosis and vitiligo, which might be related to mitochondrial damage and overactive innate immune response [18,72]. Prebiotics and probiotics theoretically maintain the balance of this axis by enhancing the health of normal intestinal flora and introducing additional beneficial microorganisms, respectively [106]. Examples of prebiotics include fermented juices and dietary fibers, and probiotics are dried bacterial capsules [107,108]. There are also combination supplements containing both prebiotics and probiotics (known as symbiotics) [109]. Several probiotic strains have demonstrated potential in improving immune tolerance and reducing oxidative stress, which are key factors in vitiligo. Strains such as *Lactobacillus rhamnosus GG*, *Lactobacillus plantarum*, and *Bifidobacterium bifidum* have been studied for their ability to modulate cytokine production, enhance gut barrier integrity, and reduce systemic inflammation. *L*. *rhamnosus GG* promotes regulatory T-cell activity, potentially reducing autoimmunity linked to vitiligo [110]. *L*. *plantarum* has antioxidant properties that mitigate oxidative stress markers in circulation, a key driver of melanocyte destruction [111]. *B. bifidum* enhances gut barrier function, limiting systemic inflammation that can exacerbate vitiligo progression [112]. While these preliminary findings are promising, larger, long-term trials are necessary to confirm the efficacy and safety of microbiome-targeted therapies. Incorporating probiotics and prebiotics as part of an integrative approach to vitiligo management holds potential, but more robust clinical data are required to establish standardized protocols. At the time of writing, however, no studies have clinically assessed the effectiveness of any of these supplements in vitiligo patients.

## 5. Plant-Derived Compounds

**Figure 3:**
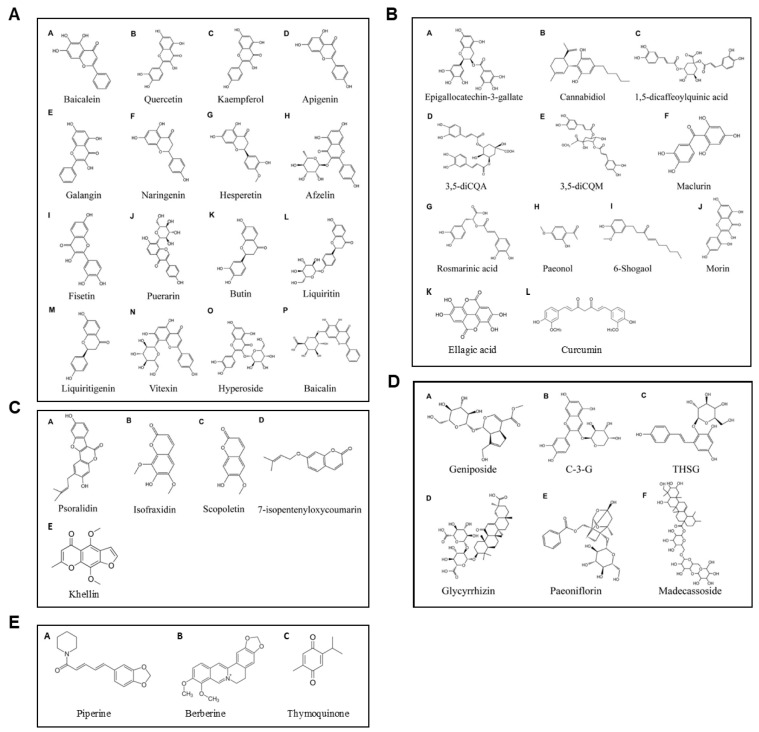
Chemical structures of plant-derived compounds with promise for vitiligo management. Flavonoids: (**AA**) Baicalein, (**AB**) Quercetin, (**AC**) Kaempferol, (**AD**) Apigenin, (**AE**) Galangin, (**AF**) Naringenin, (**AG**) Hesperetin, (**AH**) Afzelin, (**AI**) Fisetin, (**AJ**) Puerarin, (**AK**) Butin, (**AL**) Liquiritin, (**AM**) Liquiritigenin, (**AN**) Vitexin, (**AO**) Hyperoside, and (**AP**) Baicalin; phenolic compounds: (**BA**) EGCG, (**BB**) Cannabidiol, (**BC**) 1,5-dicQA, (**BD**) 3,5-diCQA, (**BE**) 3,5-diCQM, (**BF**) Maclurin, (**BG**) Rosmarinic acid, (**BH**) Paeonol, (**BI**) 6-Shogaol, (**BJ**) Morin, (**BK**) Ellagic acid, and (**BL**) Curcumin; coumarins: (**CA**) Psoralidin, (**CB**) Isofraxidin, (**CC**) Scopoletin, (**CD**) 7-isopentenyloxycoumarin, and (**CE**) Khellin; glycosides: (**DA**) Geniposide, (**DB**) C-3-G, (**DC**) THSG, (**DD**) Glycyrrhizin, (**DE**) Paeoniflorin, and (**DF**) Madecassoside; and other highlighted compounds: (**EA**) Piperine, (**EB**) Berberine, and (**EC**) Thymoquinone. Adapted from [153].

Plant-derived compounds have shown promise in vitiligo management owed to their diverse phytochemical compositions, including antioxidants, anti-inflammatory agents, and melanogenic stimulators. These compounds, their active ingredients, potential benefits, and precautions and/or interactions, are summarized in Table 3. Their chemical structures are illustrated in Figure 3.

**Table 3:** Plant-derived compounds.

Compound	Active Ingredient	Potential Benefits	Precautions/Interactions	References
*Ginkgo biloba*	Polyphenols, flavonoids (e.g., naringenin, hesperetin, baicalein)	-Decreases oxidative stress -Supports melanogenesis	-May increase risk of bleeding -May reduce effects of alprazolam, anticonvulsants, statins	[113,114,115,116]
*Polypodium leucotomos*	Phenolic compounds	-Reduces UV-induced oxidative stress -Supports repigmentation	Rare gastrointestinal discomfort	[117,118,119]
Khellin	Furanochromone	Stimulates melanocytes when combined with UVA light	Rare photosensitivity when used with UV therapy	[120,121]
*Pyllanthus embelica*	Phenolic compounds, terpenoids, amino acids, alkaloids, and vitamins	Reduce UVB-induced keratinocyte inflammation and apoptosis	-May increase risk of bleeding -May decrease blood sugar	[122,123,124]
Black Pepper (*Piper nigrum*)	Piperine	Stimulates melanocytes when combined with UVB light	May cause gastrointestinal discomfort	[16,125]
*Nigella sativa*	Thymoquinone	Decreases generation of highly reactive oxygen species	May increase risk of bleeding	[126,127,128]
Pomegranate (*Punica granatum*)	Ellagic acid, flavonoids	-Regulates T-cell function -Antioxidant and anti-inflammatory effects	-May increase risk of bleeding -May reduce effects of statins, carbamazepine -May increase effects of CYP2C9 substrates	[129,130,131]
Green tea (*Camellia sinensis*)	Epigallocatechin-3-gallate	Modulates inflammatory cytokine release	May reduce effects of statins, nadolol	[132,133,134,135]
Turmeric (*Curcuma longa*)	Curcumin	Reduces inflammatory mediator production	-May decrease blood sugar -May reduce effects of certain medications (e.g., talinolol)	[136,137,138]
*Psoralea corylifolia*	Psoralens (e.g., isopsoralen, imperatorin)	-Reduces inflammation -Promotes pigmentation with UV exposure	-Increases risk of phototoxicity -May interact with CYP3A4 substrates	[139,140,141,142]
*Melissa officinalis* (Lemon Balm)	Rosmarinic acid	-Promotes melanogenesis -Reduces UV-induced oxidative stress	-	[143,144]
*Berberis vulgaris* (Barberry)	Berberine	-Promotes melanogenesis (production and dispersion)	-Potential for gastrointestinal discomfort	[145,146,147]
Licorice (*Glycyrrhiza glabra*)	Glycyrrhizin, terpenoids	-Antioxidant and anti-inflammatory properties	-May increase risk of bleeding -Interactions with immunosuppressants	[148,149,150,151,152]

### 5.1. Ginkgo Biloba

- Primary phytochemicals: Polyphenols, flavonoids

*Ginkgo biloba* extract, a rich source of polyphenols and flavonoids, holds significant promise for vitiligo management due to its antioxidant and immunomodulatory properties [154,155,156]. Flavonoids in *G. biloba*, such as naringenin, hesperetin, and baicalein, not only act as antioxidants but also enhance melanin production by stimulating melanogenic pathways. For instance, naringenin has been shown to activate the Wnt-β-catenin signaling pathway, which increases the expression of melanogenic enzymes and may support repigmentation in vitiligo.

*G. biloba* extract reduces oxidative stress by decreasing macrophage oxidative bursts, thereby limiting the generation of reactive oxygen species [157]. Activation of the Nrf2-ARE pathway by *G. biloba*, particularly the EGb761 extract, appears to play a role in protecting melanocytes from oxidative damage [158]. In a double-blind placebo-controlled study including 25 vitiligo patients, those administered 40 mg *G. biloba* extract three times daily experienced a significant cessation of depigmentation progression (*p* = 0.006) compared to controls receiving placebo doses [159]. A prospective open-label trial of 60 mg of standardized *G. biloba* twice daily for 12 weeks in individuals ages 12 to 35 (*n* = 12) demonstrated a total VASI improvement from 5.0 to 4.5 (*p* =0.021) [155]. Further, vitiligo progression was halted in all patients with an average of 15% for repigmentation of vitiligo lesions across all participants (*p* < 0.001). The safety profile appears favorable, with few reported side effects, including headache or gastrointestinal upset. Yet, it is important to inform patients that these interventions are not yet established treatments and are considered part of ongoing research rather than standard therapy.

### 5.2. Polypodium Leucotomos

- Primary phytochemicals: Phenolic compounds

*Polypodium leucotomos* extract acts as a chemophotoprotector agent against PUVA-induced skin phototoxicity through an antioxidant-mediated effect [160]. In a randomized double-blind placebo-controlled trial, participants who received *P. leucotomos* three times daily combined with narrow-band UVB treatment twice weekly for 25–26 weeks (*n* = 50) showed increased repigmentation on the head and neck compared to the placebo group, with a borderline significant difference (*p* = 0.06) [161]. Another randomized study demonstrated that oral *P. leucotomos* supplementation alongside NB-UVB therapy (*n* = 23) for generalized vitiligo resulted in a significantly higher repigmentation rate compared to NB-UVB alone (*n* = 21) (47.8% vs. 22%, *p* < 0.0005) [162]. This herb is commonly well-tolerated, but gastrointestinal and/or pruritus-related side effects may occur in rare cases. In a broad review of 19 randomized controlled trials, mild-to-moderate gastrointestinal complaints and/or pruritus were reported in 2% (16/1016) of patients [117]. Individual studies involving administration of 480 mg *P. leucotomos* for 60 days (*n* = 20) and 240–480 mg *P. leucotomos* for 6 months (*n* = 45) reported zero or fewer (vs. placebo) adverse events, respectively [163,164]. The incidence of adverse events secondary to *P. leucotomos* usage in vitiligo patients, specifically, has not been evaluated.

### 5.3. Khella (Ammi Visnaga)

- Active phytochemical: Khellin

Khellin, when activated by UVA, stimulates melanocyte proliferation and melanogenesis [120]. In an open-label 1-year prospective study of combination khellin and 308 nm excimer laser treatment, 20 patients with resistant vitiligo showed >75% repigmentation in 45% (9/20) and 50–75% repigmentation in 25% (5/20) [165]. There was greater repigmentation in patients with shorter disease duration (<2 y, *p* = 0.02) and greater number of treatment sessions (>24, *p* = 0.03). Khellin offers an advantage as a less phototoxic alternative to psoralen in phototherapeutic regimens [166,167].

### 5.4. Phyllanthus Emblica (Amla or Indian Gooseberry)

- Active phytochemicals: Antioxidants and anti-inflammatory compounds

The anti-inflammatory effects of Phyllanthus emblica have been harnessed for the treatment of diseases such as pneumonia, hepatitis, and cancer [168]. In an *ex vivo* study assessing its use against UVB-induced keratinocyte inflammation and apoptosis, Phyllanthus emblica (PE) demonstrated antioxidative, anti-inflammatory, and anti-apoptotic effects in UVB-exposed HaCaT cells, with dose-dependent reductions in ROS levels and apoptotic cell death (*p* < 0.05) [122]. At 100 µg/mL, PE increased catalase activity by 30%, reduced phosphorylation of c-Jun and NF-κB activation, and lowered PGE2 production by over 75% from UVB-induced levels. Additionally, PE reduced Akt phosphorylation from 1.42 to 0.63-fold, underscoring its protective effects against oxidative stress, inflammation, and apoptosis. A clinical trial of 65 vitiligo patients treated with an oral supplement (*P. emblica* fruit extract 100 mg, vitamin E 4.7 mg, and carotenoids 10 mg) three times daily for 6 months, alongside 65 controls, both receiving topical therapy and/or phototherapy, found that the supplement group had higher rates of mild repigmentation (1–25%) of the head and neck (*p* = 0.019), reduced inflammation (*p* = 0.002), and slower lesion progression (*p* = 0.039) [105]. Moreover, rates of non-repigmentation after 6-month follow-up were significantly higher in the control population at all tested body sites. Currently, the limited number of large clinical trials restricts clinical recommendation for vitiligo, but its positive safety profile and efficacy in preliminary reports makes *P. emblica* a strong candidate for future investigation.

### 5.5. Black Pepper (Piper Nigrum)

- Active phytochemical: Piperine

Piperine has demonstrated anti-inflammatory, antiarthritic, and antinociceptive effects in animal models [169]. In a double-blind randomized clinical trial, 63 facial vitiligo patients were randomly assigned to either the piperine group (*n* = 35) or the placebo group (*n* = 28, 44.4%), and both groups adjunctly received UVB phototherapy every other day for 3 months [170]. After one month of treatment, burning sensations of the treated area were reported in 10/35 patients (28.6%) treated with piperine but none of the controls (*p* = 0.002), and skin redness affected 6/35 patients (17.1%) in the piperine group compared to none in the control group (*p* = 0.028). At the conclusion of the trial, based on the average of two dermatologists’ assessments, patients receiving adjunct piperine reported significantly greater repigmentation compared to the placebo group (mean 53.6% [SD 13.4%] vs. 16.3% [7.2%], *p* < 0.001). Gastrointestinal side effects were not reported in this study but have been observed (i.e., abdominal pain, gastric bleeding) in previous piperine-based trials [171,172]. While the compound shows promise, further randomized controlled trials are required to establish standardized dosing protocols and ensure its safety in long-term use.

### 5.6. Nigella Sativa

- Active phytochemical: Thymoquinone

Thymoquinone, the primary component found in *Nigella sativa* seeds, shields organs from oxidative harm caused by various pathologies that generate free radicals [173]. In an Iran-based randomized double-blind clinical trial, 52 patients with vitiligo applied either topical *N. sativa* oil or fish oil to their lesions twice daily for 6 months [174]. The *N. sativa* group showed a significant reduction in VASI, with mean scores decreasing from 4.98 to 3.75 (r = 0.864, *p* = 0.02). In contrast, the fish oil group experienced a smaller nonsignificant reduction, with mean VASI scores decreasing from 4.98 to 4.62 (r = −0.489, *p* = 0.067). Patients did not note any side effects over the trial duration. Moreover, a Turkish study assessed the efficacy of a topical *N. sativa*-containing cream (ABRAGEN™ Tone Regulating Cream, Biota Botanical Laboratories, İstanbul, Turkey) in 33 vitiligo patients (mean age 31.9 years) who had not received topical or systemic treatment within the previous 3 months [175]. Results indicated ≥50% repigmentation in 43.5% (10/23) of patients with facial lesions, 43.8% (7/16) of patients with hand lesions, and 87.5% (7/8) patients with genital lesions. Notably, the safety of *N. sativa* has been established across multiple randomized controlled trials undertaken in various other conditions [176,177,178]. Nonetheless, the long-term safety and efficacy of *N. sativa* require further investigation, particularly in larger patient cohorts.

### 5.7. Pomegranate (Punica Granatum)

- Active phytochemicals: Ellagic acid, flavonoids

Ellagic acid, the major component of *Punica granatum*, has shown promising immunomodulatory effects through the regulation of T-cell function and suppression of humoral immunity [179]. Used extensively in Ayurvedic medicine, Punica granatum formulations have demonstrated antioxidant activity [180,181] and anti-inflammatory activity [182]. Although promising for several dermatologic conditions, i.e., acne vulgaris and psoriasis, there have been no clinical trials specifically investigating the efficacy of *Punica granatum* in treating vitiligo to date. Additionally, standardized dosing regimens have not been well established.

### 5.8. Green Tea (Camellia Sinensis)

- Active phytochemical: Epigallocatechin-3-gallate

Epigallocatechin-3-gallate (EGCG) is a prominent compound in green tea with anti-inflammatory and immunomodulatory properties [183]. A randomized controlled trial involving 46 patients who had not recently received topical or systemic treatment (i.e., in the previous 4 or 8 weeks, respectively) found no significant difference in VASI score reduction between lesions treated twice daily with topical EGCG 3% cream and those treated with topical pimecrolimus 1% cream after 6 months (1.19 ± 0.42 to 0.63 ± 0.38 vs. 1.18 ± 0.43 to 0.61 ± 0.36; *p* = 0.755) [184]. No serious side effects were reported. Its comparison with other antioxidants will be valuable for optimizing regimens.

### 5.9. Turmeric (Curcuma Longa)

- Active phytochemical: Curcumin

Curcumin, a compound found in turmeric, exerts anti-inflammatory effects by regulating multiple signaling pathways such as NF-κB, MAPK, AP-1, and NLRP3 inflammasome, leading to the inhibition of inflammatory mediator production [185]. In a randomized, double-blind, placebo-controlled study of 30 enrolled patients aged 8–65 years with multiple discrete vitiligo lesions, 24 completed the trial (6 refused to continue), applying either turmeric or placebo cream to their lesions twice daily for four months; results showed that turmeric cream significantly reduced the size and improved the appearance of vitiligo lesions compared to placebo (*p* < 0.001) [186]. Curcumin is generally considered safe, though high doses may cause gastrointestinal issues.

### 5.10. Psoralea Corylifolia (Cullen Corylifolium)

- Active compound: Psoralens

*Psoralea corylifolia* exhibits anti-inflammatory effects attributed to compounds, including psoralen, isopsoralen, imperatorin, bavachinin A, corylin, and bakuchiol, which modulate various inflammatory pathways and reduce the release of inflammatory factors, as demonstrated in studies on ulcerative colitis, rheumatoid arthritis, liver inflammation, and myocardial inflammation [187]. In a self-controlled trial with 20 vitiligo patients ages 18–60, daily application of a formulated hydrophilic ointment containing 10% *w*/*w P. corylifolia* seed powder on selected white lesions for 12 weeks led to a statistically significant increase in pigmentation compared to untreated lesions on the same patients (*p* < 0.05), indicating the ointment’s potential efficacy as a monotherapy for vitiligo [142]. Psoralens are known to increase the risk of phototoxicity, so monitoring and appropriate dosing are crucial. Head-to-head comparisons with other photo-sensitizers like khellin could provide insights for optimized treatment.

### 5.11. Melissa Officinalis (Lemon Balm)

- Active phytochemical: Rosmarinic acid

Extracts from *Melissa officinalis* have been identified as melanogenic activators due to their phenolic compounds, such as Rosmarinic acid. These substances can promote endogenous melanin production by upregulating tyrosinase and other melanogenic enzymes. An in vitro experimental study evaluating the protective effect of lemon balm extract and Rosmarinic acid found that lemon balm extract significantly (*p* < 0.05) enhanced keratinocyte survival, reduced UVB-induced ROS production and DNA damage, and promoted melanogenesis compared to untreated cells, indicating its potential as a photoprotective agent against UVB-induced skin damage [143]. Future studies exploring the mechanisms of *M. officinalis* involved in melanogenesis and their potential efficacy as pigmenting agents are warranted.

### 5.12. Berberis Vulgaris (Barberry)

- Active phytochemical: Berberine

Berberine, a plant alkaloid found in Berberis vulgaris, possesses a wide variety of pharmacological and biological properties due to its antimicrobial, anti-inflammatory, and antioxidative effects [188]. These properties of berberine suggest its potential to protect melanocytes by inhibiting autoimmune destruction, thereby preventing depigmentation and promoting the repigmentation of vitiligo patches [189]. An in vitro experimental study investigating the ultrastructural effects of berberine on the melanophores of *Bufo melanostictus* to assess its skin-darkening potential revealed that berberine significantly (*p* < 0.05) increased the number of melanophores and mature melanosomes in a dose-dependent process, suggesting its potential as a melanogenic agent [145]. More studies are needed to elucidate the safety profile of berberine and strengthen its clinical applicability for the treatment of vitiligo.

### 5.13. Licorice (Glycyrrhiza Glabra)

- Primary phytochemicals: Terpenoids (glycyrrhizin)

Terpenoids such as glycyrrhizin, derived from *Glycyrrhiza glabra*, can stimulate melanogenesis by elevating intracellular cyclic adenosine monophosphate levels. A recent meta-analysis of 39 randomized controlled trials (*n* = 3994) found that the addition of glycyrrhizin to conventional vitiligo therapies had superior repigmentation rate benefits compared to phototherapy alone (RR = 1.28, 95% CI: 1.21–1.36, *p* < 0.001, *I*^2^ = 0%; 15 trials, *n* = 1993) and immunosuppressant therapy alone (RR = 1.76, 95% CI: 1.49–2.08, *p* < 0.001, *I*^2^ = 41%; 6 trials, *n* = 483) [148]. Compared to combination phototherapy and immunosuppressant therapy, add-on glycyrrhizin was associated with a greater likelihood of achieving >50% repigmentation (7 studies, RR = 1.42; *p* < 0.001, *I*^2^ = 47%; 7 trials, *n* = 602). Additionally, glycyrrhizin did not significantly increase the rate of adverse events based on 26 studies with 2734 patients (RR = 0.79, 95% CI: 0.62–1.00, *p* = 0.05, *I*^2^ = 47%). Results from a Chinese open-label randomized trial found that a combination oral compound glycyrrhizin (OCG, 20 mg glycyrrhizin, 25 mg aminoacetic acid and 25 mg methionine) and UVB was superior to OCG or UVB alone, attaining a repigmentation rate of 87.5% (42/48) versus 77.1% (37/48) and 75% (36/48), respectively [149]. Facial edema was noted in 2 patients (1 receiving OCG monotherapy and 1 receiving OCG plus UVB combination therapy), but symptoms resolved without treatment.

## 6. Patient Counseling

Patient counseling plays a pivotal role in the successful implementation of dietary and plant-based interventions for vitiligo. In real-world settings, dietary therapies should be introduced as complementary measures rather than as substitutes for standard treatments. Physicians can recommend incorporating antioxidant-rich foods into daily meals and potentially avoiding inflammatory foods. Suggested antioxidant-rich foods include apples, green tea, Indian gooseberry, onions, and peppers—each of which contains bioactive compounds that may counteract oxidative stress [16,190]. Regarding inflammatory foods, multiple case reports have reported improvement in vitiligo symptoms after removing gluten from patient diets [67,68]. The complementary approach of avoiding foods that contribute to oxidative stress while incorporating anti-inflammatory foods is also important. This means counseling patients to refrain from mango, certain nut types, and several berry varieties given their high phenol content, while simultaneously encouraging seafood given its high omega-3 fatty acid content [191,192]. Other possible therapeutic food suggestions include those high in carotenoids (i.e., bell peppers, carrots, pumpkin, tomato products) and Cremini mushrooms given their UV-protective effects and vitamin contents, respectively [192,193]. At the same time, however, it is important to note that data supporting these dietary recommendations are lacking [194]. Early data suggest that these interventions are generally safe, but concurrent use with existing treatment paradigms requires further evaluation in a controlled setting to avoid unanticipated adverse effects or efficacy reduction. Accordingly, physicians can continue to emphasize how dietary modifications are largely adjunctive at this time and ensure patients prioritize traditional treatments (i.e., phototherapy) and continue avoiding foods to which they are hypersensitive [195].

In implementing these dietary practices, dermatologists play a critical role alongside dieticians. Dermatologists serve as the medical expert on vitiligo in these interdisciplinary care teams [196]. They may consequently counsel patients on the nature of vitiligo, mechanism of action of nutritional interventions, patient-specific eligibility in the context of other comorbidities, and realistic expectations of treatment outcomes. Throughout treatment, they may also counsel patients on what measures are being used to track response to therapy or lack thereof. Dietitians, on the other hand, bring expertise in personalized nutrition by developing patient-specific meal plans based on the dermatologist’s guidance. They can also adapt these plans dynamically in response to patient preferences, nutritional needs, and any food allergies [197]. 

Frequent communication between dermatologists and dietitians/nutritionists ensures a cohesive approach and reinforces adherence. Clinicians should frame dietary and plant-derived interventions as adjunctive measures with emerging but incomplete evidence. Transparent communication about the experimental nature, potential benefits, and limitations of these therapies will help manage patient expectations and build trust.

## 7. Practical Implications and Recommendations

At the time of writing, primary treatments for vitiligo include topical anti-inflammatories, phototherapy, and systemic corticosteroids [198]. For cases that are extensive or unresponsive yet stable, transplantation surgery may be considered, while Janus kinase inhibitors are an emerging option with promising trial results [199,200,201]. However, treatment-resistant cases highlight a potential role for supportive measures such as dietary supplements and natural compounds to potentially enhance standard therapies and patient outcomes [202].

Incorporating a diet rich in antioxidants shows potential benefits in reducing oxidative stress, which is increasingly recognized as a factor in the pathogenesis of vitiligo [20]. Certain nutrients with anti-inflammatory and immunomodulatory properties are recommended for their possible roles in supporting skin health and immune regulation [17]. While existing studies provide valuable preliminary insights into the impacts of dietary modifications on vitiligo, robust evidence from large-scale clinical trials is needed to establish standardized protocols for dietary interventions. Combined with conventional treatments, these dietary strategies may offer adjunctive support, potentially enhancing repigmentation outcomes and reducing the frequency of disease flares [104]. Therefore, physicians might consider integrating nutritional counseling within a comprehensive management plan, particularly for patients with refractory or severe forms of vitiligo, while clearly positioning these interventions as complementary to primary medical therapies.

## 8. Future Directions and Priorities

A significant limitation of the current evidence base and, relatedly, forward progress, is the lack of large-scale controlled trials evaluating the efficacy of dietary and plant-based therapies in vitiligo. It is necessary for funding bodies to recognize the potential translational impact of such studies in optimizing patient care. In the interim, physicians must approach these interventions cautiously and prioritize evidence-based practices.

Currently, standard treatments for vitiligo largely focus on the autoimmune aspects of the disease through immunomodulatory strategies (e.g., UV radiation, corticosteroids). However, limited success with these approaches for specific patients suggests a complementary role for dietary supplements and plant-derived adjuncts to potentially enhance treatment efficacy. To continue advancing nutritional interventions as a promising and trusted adjunct for managing vitiligo, future investigations should tackle several critical areas (Table 4).

Emerging technologies in dietary assessment, including mobile apps and wearable devices, offer promise for precise, real-time tracking of patients’ nutritional intake [202]. Integrating AI could further personalize dietary interventions based on individual multi-omic profiles and dietary behaviors. Collectively, advances in these areas can help structure long-term studies to clarify causal relationships between diet and vitiligo progression.

Alongside tech-driven interventions, the early successes of plant-derived compounds, such as *Ginkgo biloba*, warrant additional study. Likewise, research has increasingly highlighted vitamins and trace minerals as vital components in the management of vitiligo given their role in immune modulation, melanogenesis, and antioxidative defense. Comprehensive, controlled trials may not only clarify their individual benefits but also reveal synergistic effects when combined with dietary and phototherapeutic strategies, in addition to defining implicated biological pathways, dosing standards, and safety.

The gut–skin axis represents another frontier, with a focus on the potential of prebiotics, probiotics, and synbiotics to modulate immune responses and support skin health. Research increasingly suggests that a balanced gut microbiome may play a key role in immune regulation and melanocyte protection, potentially influencing vitiligo progression.

Achieving these aims will require robust collaboration between dermatologists and nutritionists to ensure care that addresses both skin health and nutritional needs. By fostering interdisciplinary partnerships and leveraging innovative assessment tools, future research can significantly advance our understanding of the interplay between nutrient intake and vitiligo, ultimately leading to more effective management strategies.

## 9. Conclusions

While standard therapies (e.g., phototherapy, topical corticosteroids, JAK inhibitors) have demonstrated efficacy for managing vitiligo, the persistent and recurrent nature of the disease underscores the need for supplementary strategies. As a supplement, dietary strategies offer a promising but experimental approach to modulate oxidative stress and support immune balance. The potential benefits of vitamins (C, E, B12, folate), trace elements (zinc, copper), and antioxidant-rich foods may also enhance treatment efficacy, yet these remain adjunctive to traditional therapies. Moreover, phytochemicals demonstrate protective effects on melanocytes, reducing oxidative stress and modulating immune responses. High-fat diets exacerbate and low-refined carbohydrate, high-protein diets potentially improve disease outcomes by regulating autophagy and oxidative stress. Also notable, the potential of probiotics to influence the gut-skin axis suggests therapeutic value through microbial modulation. However, the data supporting these recommendations are still preliminary, necessitating further large-scale clinical studies to establish true efficacy and, later, standardized protocols. Integrating nutritional counseling into vitiligo management, with support from dietitians, could foster a more holistic approach. Emphasis should continue to be placed on ensuring that these interventions complement primary therapies, stressing that ongoing research may substantiate, refine, or reframe these preliminary recommendations in the future.

## Figures and Tables

**Table 1:** Dietary strategies for treatment of vitiligo.

Nutrient/Compound	Findings	Studied Patient Population(s)
Vitamin D	Lower levels; higher IL-17; adjunct therapy reduces lesion size; positive correlation with disease duration	Males, younger, non-UV treated
Vitamins C and E	Mixed serum level results; no significant differences in meta-analysis	Non-specified vitiligo
Selenium	Controversial findings; generally lower levels in recent studies; consistent in meta-analyses; lower in Asians	Asian vitiligo patients
Copper and Zinc	Conflicting results; lower Cu in tissue; different serum levels in recent studies	Vitiligo patients vs. controls
Baicalein, Quercetin, etc.	Protect melanocytes, have antioxidant effects, promote repigmentation; antioxidative and anti-inflammatory	Non-specified vitiligo
Saturated Fat, PUFAs	Higher SFA intake and lower EPA and DHA associated with higher BMI and VASI; increased risk with high total fat intake	Vitiligo hx < 5 y; adult nonsegmental vitiligo
Refined Carbohydrates	High-carb diets linked to dysregulated autophagy, higher blood sugar, oxidative stress; correlation with high VASI, hyperglycemia, ROS generation	Early-stage and stabilized vitiligo; adult nonsegmental vitiligo; non-specified vitiligo
Gluten-Free Diet	Effective for resistant vitiligo; associated with celiac disease; progressive and sustained repigmentation; higher CD incidence	Vitiligo patients with celiac disease
Gut Microbiome	Reduced diversity and richness; altered bacterial abundance; decreased Bacteroidetesratio; distinct microbial composition	Advanced, unstable, and non-specified nonsegmental vitiligo

**Table 2:** Primary nutritional supplements, associated mechanisms of action, and level of evidence supporting or dissuading their use in management of vitiligo.

Nutritional Supplement	Mechanism of Action	Highest Level of Evidence * Supporting Use	Highest Level of Evidence * Against Use
Vitamin D	Improve vitamin D levels to limit risk of autoimmunity from hypovitaminosis D	2	1
Antioxidants	Mitigate number of reactive oxygen species to limit melanocyte stress and damage	1	1
Unsaturated Fatty Acids	Limit inflammatory sequelae of dysregulated fatty acid metabolic pathways	1	None currently
Probiotics and prebiotics	Maintain gut–skin axis health to reduce mitochondrial damage and autoimmune risk	None currently	None currently

* Adapted from criteria established by the Oxford Centre for Evidence-based Medicine.

**Table 4:** Future directions for vitiligo management through nutrition and supplementation.

Priority Area	Proposed Actions
Epigenetic modulation through diet	-Track DNA methylation changes in vitiligo patients on antioxidant-rich diets -Assess diet-induced epigenetic effects on melanocyte survival
‘Intelligent’ personalized dietary interventions	-Train AI on multi-omic and dietary data -Develop real-time predictive models for dietary impact on vitiligo severity
Gut–skin axis and microbiome engineering	-Test targeted prebiotics and probiotics for immune modulation in vitiligo -Map metabolite effects on melanocyte function and optimize diets to boost beneficial metabolites -Personalize synbiotics using patient-specific microbiome data
Psycho-dietary research integration	-Assess combined anti-inflammatory diets and stress reduction for vitiligo control -Investigate brain–gut–skin effects on diet response
Nutrient biomarkers for early detection/action	-Identify biomarker panels for dietary responsiveness in vitiligo -Develop point-of-care tests for real-time nutrient adjustments -Establish lipidomic markers linked to melanocyte protection
Integrative digital platforms for engagement	-Integrate wearable data (i.e., UV exposure, activity) for holistic lifestyle tracking
Cross-cultural dietary studies	-Compare diet-specific inflammatory and antioxidant profiles globally -Develop dietary guidelines from multi-country cohort findings
Biochemical pathway exploration of phytochemicals	-Map phytochemical effects on melanogenesis via metabolomics -e.g., test phytochemical synergy with JAK inhibitors and phototherapy
